# Hereditary Congenital Cataract With Spastic Paraplegia Associated With a Missense PAX6 Variant (p.Arg26Trp)

**DOI:** 10.7759/cureus.115197

**Published:** 2026-08-25

**Authors:** Shiroh Miura, Koji Namiguchi, Sayaka Matsumoto, Yinrui Sun, Hiroki Shibata

**Affiliations:** 1 Department of Neurology and Geriatric Medicine, Ehime University Graduate School of Medicine, Toon, JPN; 2 Department of Ophthalmology, Ehime University School of Medicine, Toon, JPN; 3 Division of Genomics, Medical Institute of Bioregulation, Kyushu University, Fukuoka, JPN

**Keywords:** autosomal dominant, congenital cataract, hereditary spastic paraplegia, missense, nonsynonymous, pax6

## Abstract

To date, several genetic factors have been reported in cases presenting both congenital cataract (CC) and spastic paraplegia (SPG), but the overall genetic landscape of this condition remains insufficiently understood. Here, we describe the clinical, genetic, and functional findings in a single Japanese family presenting with hereditary CC and SPG. Affected individuals exhibited severe CC and mild SPG symptoms. Whole exome sequencing combined with stringent filtering revealed no pathogenic variants in the three known genes. Instead, we identified a heterozygous missense variant in *PAX6* (NM_001258465, c.76C>T: p.Arg26Trp), a gene well known for its role in CC. RNA expression analysis showed no significant difference in *PAX6* transcript levels between affected individuals and healthy controls. However, luciferase reporter assays demonstrated that the PAX6 variant protein exhibited significantly increased reporter activity compared to the wild-type protein. These findings indicate that the phenotypic spectrum of PAX6 may extend to include SPG, as observed in this single family, potentially through altered transcriptional activity.

## Introduction

Congenital cataract (CC) is an ophthalmic disorder characterized by lens opacity present at birth or during the first year of life and can cause severe visual impairment and amblyopia [1]. It is estimated that 8.3-25% of CC cases are hereditary [2]. Hereditary spastic paraplegias (SPGs) comprise a group of monogenic neurodegenerative disorders characterized by slowly progressive lower limb spasticity and muscle weakness and are clinically classified into pure and complex forms. Complex forms are accompanied by additional neurological or systemic manifestations. Their global prevalence ranges from 0.1 to 9.6 per 100,000 individuals [3]. To date, only three genes (*ALDH18A1*, *FAR1*, and *GBA2*) have been implicated in inherited CC with SPG [4-7]. Because patients often see separate ophthalmology and neurology specialists, hereditary cases presenting with both CC and SPG may be underrecognized. We conducted genetic analyses, including whole‑exome sequencing, in a Japanese family with hereditary CC and SPG.

## Case presentation

**Figure 1:**
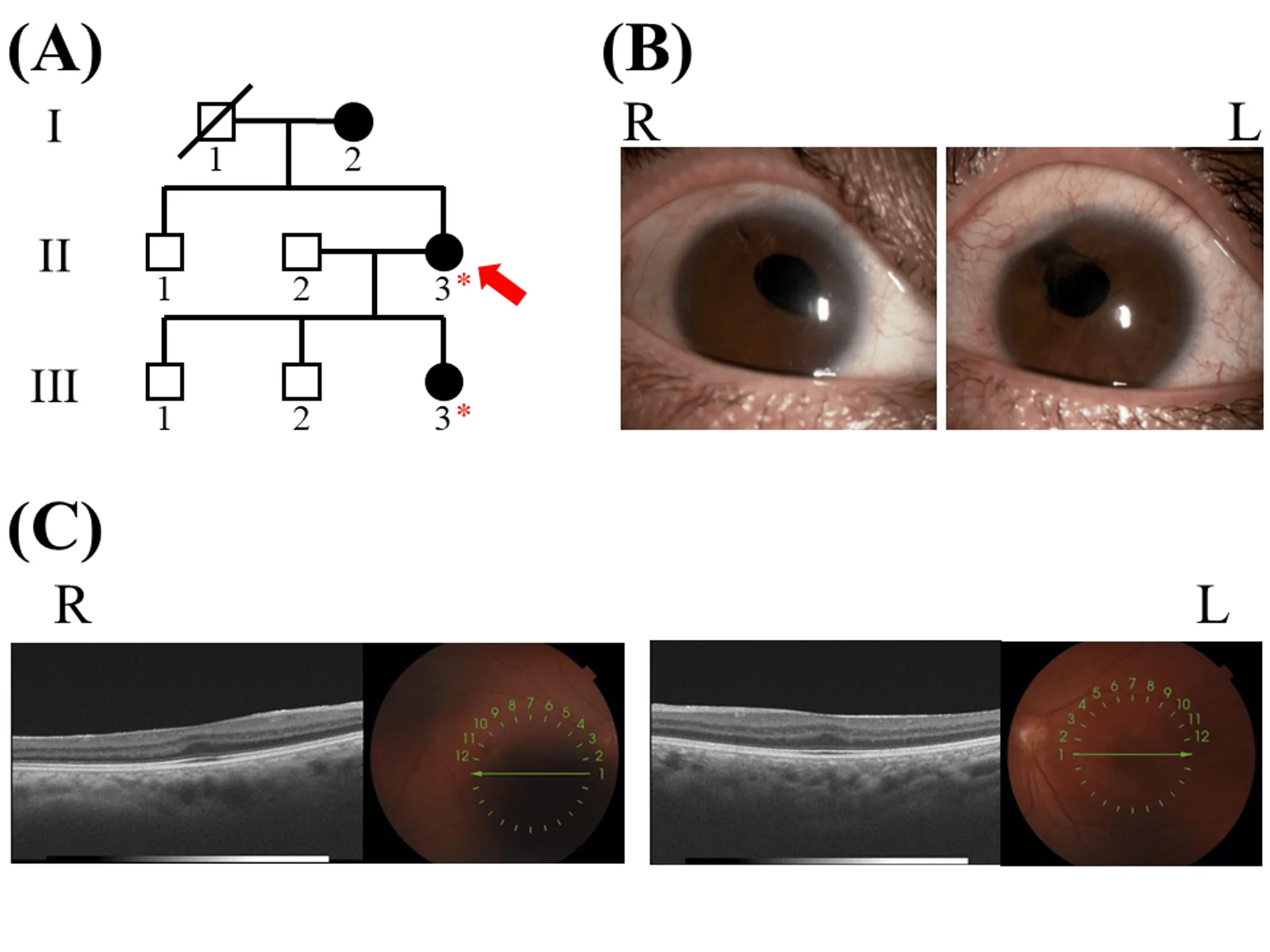
Pedigree and ophthalmic imaging. (A) Family pedigree (partial omissions are to protect the identities of other family members). Open square: unaffected male, filled circle: affected female, diagonal line: deceased, asterisk: neurologically examined and blood sampled. The proband (II-3) is indicated with a red arrow. (B) Anterior ocular segment images. Corectopia and aphakia due to cataract surgery were observed. Aniridia, corneal opacity, and corneal epithelial stem-cell deficiency were not observed. R: right, L: left. (C) Optical coherence tomography. The inner retinal layers traverse the foveal center, with absence of the physiological foveal depression and foveal bulge, thickening of the outer nuclear layer, and traces of a hyperreflective line adherent to the retinal surface. Central retinal thickness measurements were unavailable. R: right, L: left.

The study involved three family members across three generations (I-2, II-3, and III-3) (Figure 1A). Detailed clinical and genetic evaluations were performed in the two individuals, II-3 and III-3, whereas information regarding I-2 was obtained from the family history. The proband (II-3), a 52-year-old woman, has had CC, congenital nystagmus, and macular hypoplasia since early childhood. No intraocular lens was implanted during cataract surgery, and she remains aphakic, with persistent nystagmus and macular hypoplasia. She developed gait disturbances at age 44 and has comorbid diabetes mellitus, hypertension, and dyslipidemia. She completed junior high school but did not pursue further education. Neurological examination revealed pendular nystagmus without diplopia. Mild muscle weakness and decreased superficial sensation were noted, predominantly on the right side. Hyperreflexia was present in all four extremities, along with spasticity of the lower limbs, slightly more pronounced on the right. Babinski reflex was absent. There were no signs of urinary dysfunction or cerebellar ataxia. Serum anti-HTLV-I antibody was negative. Cerebrospinal fluid analysis was normal. Brain MRI showed no abnormalities, including no evidence of thin corpus callosum, and cervical MRI and single-photon emission computed tomography of the brain were normal. The patient's uncorrected visual acuity (UCVA) was 20/133 (0.82 logMAR) in the right eye and 20/100 (0.70 logMAR) in the left eye. The best-corrected visual acuity (BCVA) was 20/100 (0.70 logMAR) in the right eye with a correction of +0.50 spherical diopters (D) and −1.00 cylindrical D at axis 140°, and 20/67 (0.52 logMAR) in the left eye with a correction of −1.00 spherical D and −1.00 cylindrical D at axis 170°. Anterior ocular segment imaging revealed corectopia and aphakia following cataract surgery (Figure 1B). No coloboma was observed in any region of the eye. Optical coherence tomography demonstrated macular hypoplasia, characterized by persistence of the inner retinal layers across the foveal center, absence of the physiological foveal depression and foveal bulge, thickening of the outer nuclear layer, and traces of a hyperreflective line adherent to the retinal surface (Figure 1C). Central retinal thickness measurements were unavailable. The proband's mother (I-2) attended a school for the blind and had a history of diabetes mellitus and hypertension. Patient III-3, the proband's 19-year-old daughter, underwent cataract surgery in infancy and remains aphakic, with persistent congenital nystagmus and macular hypoplasia. She graduated from high school but did not pursue further education. Neurological examination showed pendular nystagmus without diplopia, hyperreflexia in all extremities, and mild bilateral equinus.

**Figure 2:**
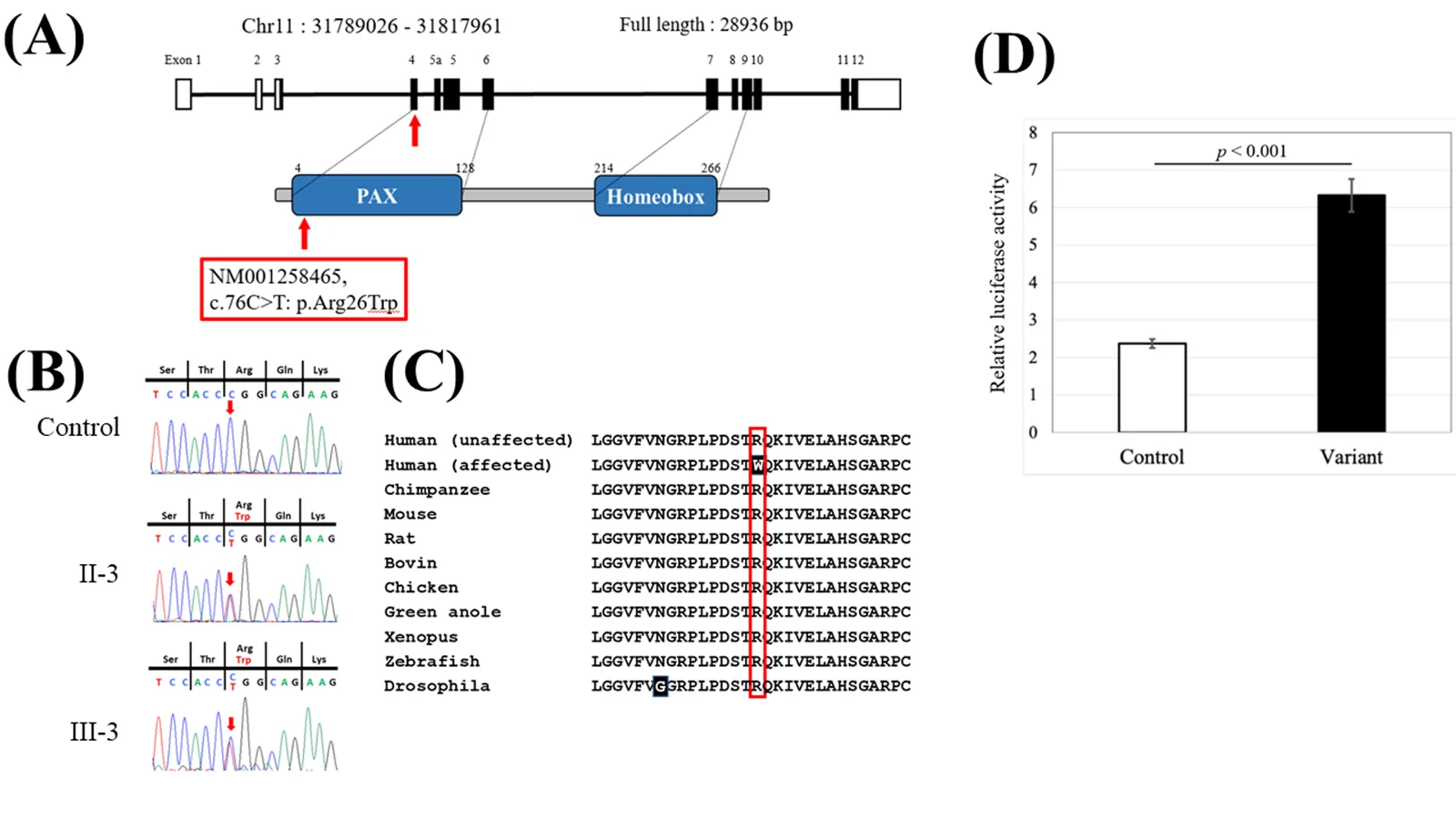
Identification and functional characterization of the PAX6 variant. (A) Diagram of the structures of the gene and domain of *PAX6.* The location of the variant is indicated by a red arrow. Coding sequences and untranslated region (UTR) sequences are shown in filled and open boxes, respectively. (B) Sanger validation of the *PAX6* variant. Electropherogram of the *PAX6* variant in an unaffected control, the proband (II-3), and her daughter (III-3). The location of the variant is indicated by a red arrow. (C) Alignment of amino acid sequences in the region containing the variant in *PAX6.* Multiple alignments of the region where the variants are located. The amino acid substitutions identified in the pedigree are shown by red boxes. Amino acids different from the human reference sequence are shown in black. (D) Luciferase activity on wild-type and variant constructs of PAX6. The relative luciferase activity of wild-type and variant PAX6 is shown. Error bars indicate standard deviation (SD). SD = 0.117 and 0.433, respectively. p = 7.93 x 10^-6^.

Whole exome sequencing of the proband (II-3) identified 24,803 single nucleotide variants, among which 21 functional variants in prioritized genes (Tables 1, 2) were detected. Filtering against public databases (1000G, ExAC, gnomAD; allele frequency >0.001%) narrowed the candidates to a single heterozygous *PAX6* exon 4 missense variant, c.76C>T (p.Arg26Trp) (Figure 2A). This variant was absent in 83 local controls and was confirmed in both affected individuals (II-3 and III-3) (Figure 2B). The substitution affects a highly conserved residue within the PAX paired domain (Figure 2C), a critical region for DNA binding and essential for transcriptional activity [8], and multiple in silico analyses consistently supported its pathogenicity. Based on American College of Medical Genetics and Genomics (ACMG)/Association for Molecular Pathology (AMP)/College of American Pathologists (CAP) criteria [9], the variant was classified as pathogenic. RNA sequencing of patients II-3 and III-3 showed no significant reduction in *PAX6* transcript levels compared with unaffected in-house controls (data not shown). Functional assessment using a luciferase reporter assay demonstrated that the p.Arg26Trp variant increased reporter activity by 2.67-fold relative to wild-type PAX6 (Figure 2D), suggesting altered transcriptional activity that may contribute to the observed phenotype.

**Table 1:** Genes associated with hereditary spastic paraplegia.

Gene	MIM number	Gene	MIM number
ABHD16A	142620	KIF5A	604187
ACP33	248900	KLC2	609541
ALDH18A1	601162	KPNA3	601892
AMFR	603243	L1CAM	312900
AMPD2	615686	MAG	616680
AP4B1	614066	MARS1	620323
AP4E1	613744	MTRFR	615035
AP4M1	612936	NIPAI	600363
AP4S1	614067	NT5C2	613162
AP5Z1	613647	PCYT2	618770
ARL6IP1	615685	PGAP1	615802
ARSI	610009	PI4KA	619621
ATL1	182600	PLP1	312920
ATP13A2	617225	PNPLA6	612020
ATP6	516060	RAB3GAP	602536
B4GALNT	609195	REEP1	610250
BSCL2	270685	REEP2	615625
C19orf12	615043	RNF170	619686
CAPN1	616907	RTN2	604805
CPT1C	616282	SELENOI	618768
CYP2U1	615030	SLC16A2	300523
CYP7B1	270800	SLC33A1	612539
DDHD1	609340	SPART	275900
DDHD2	615033	SPAST	182601
DSTYK	270750	SPG7	607259
ENTPD1	615683	SPTAN1	182810
ERLIN1	615681	SPTSSA	613540
ERLIN2	611225	TECPR2	615031
FA2H	612319	TFG	615658
FAR1	619338	TMEM63C	619953
FARS2	617046	UBAP1	618418
GBA2	614409	UCHL1	615491
GJC2	613206	USP8	603158
HPDL	619027	VPS37A	614898
HSPD1	605280	WASHC5	603563
IBA57	616451	WDR48	612167
KIAA1840	604360	ZFR	615635
KIF1A	610357	ZFYVE26	270700
KIF1C	611302	ZFYVE27	610244

**Table 2:** Genes associated with congenital cataract.

Gene	MIM number	Gene	MIM number	Gene	MIM number
ABCD3	616278	FBN1	608328	NR2E3	604485
ABHD12	612674	FKRP	613153	OAT	258870
ADAMTS18	186580	FKTN	253800	OCRL	309000
AGK	212350	FLNB	150250	PAX6	106210
AIC	304050	FOXE3	612968	PEX1	214100
ALDH18A1	616603	FTL	600886	PEX10	614870
ARSE	302950	FYCO1	610019	PEX12	614859
B3GALTL	261540	FZD4	133780	PEX13	614883
BCOR	300166	GALE	230350	PEX14	614887
BEST1	193220	GALK1	230200	PEX26	614872
BFSP1	611391	GALT	230400	PEX3	614882
BFSP2	611597	GBA2	614409	PEX5	214110
BUB1B	257300	GCM2	146200	PEX7	215100
CAV1	606721	GCNT2	116700	POLG	157640
CC2D2A	612285	GFER	613076	POMGNT1	253280
CHMP4B	605387	GJA1	164200	POMT1	236670
CLPB	616271	GJA3	601885	POMT2	613150
CNBP	602668	GJA8	116200	PQBP1	309500
CNGB3	262300	GJB6	129500	PRX	605725
COL11A1	604841	GLA	301500	PTEN	158350
COL18A1	267750	GNAS	103580	PTH	146200
COL4A1	607595	GNPAT	222765	PXMP3	614866
COL4A5	301050	GUCY2D	600179	RAB3GAP1	600118
COL7A1	226600	HCCS	309801	RAB3GAP2	212720
CRYAA	604219	HMX1	612109	RECQL4	268400
CRYAB	608810	HSF4	116800	RRAGA	612194
CRYBA1	600881	HSPG2	224410	SALL4	607323
CRYBA4	610425	IARS2	616007	SC5DL	607330
CRYBB1	611544	IKBKG	308300	SEC23A	607812
CRYBB2	601547	ITM2B	117300	SIL1	248800
CRYBB3	609741	JAM3	613730	SIPA1L3	616851
CRYGA	123660	KCNJ13	193230	SLC33A1	614482
CRYGB	615188	LARGE	613154	SRD5A3	612713
CRYGC	604307	LEMD2	212500	TDRD7	613887
CRYGD	115700	LIM2	615277	TFAP2A	113620
CRYGS	116100	LMX1B	161200	TRAPPC11	615356
CTDP1	604168	LONP1	605490	TRPM3	620253
CTPL1	Unregistered	LRP2	222448	TUBA1A	611603
CYP27A1	213700	LRP5	601813	UNC45B	616279
DHCR7	270400	LSS	616509	VCAN	143200
DMPK	160900	MAF	601088	VIM	116300
EBP	302960	MAN2B1	248500	VLDLR	224050
EPG5	242840	MIP	615274	VSX2	142993
EPHA2	116600	MIR184	613146	WDR36	609669
ERCC6	214150	MMP1	226600	WDR87	620274
ERCC8	216400	MVK	610377	WFS1	614296
ESCO2	268300	MYH9	153640	WNT3	273395
ETFDH	231680	NEU1	256550	XYLT2	605822
EYA1	601653	NF2	614872	YWHAE	247200
FAM126A	610532	NHS	302350		
FAR1	619338	NOD2	186580		

## Discussion

In this study, we describe a Japanese family presenting with CC and SPG associated with a rare heterozygous variant (c.76C>T) in exon 4 of the *PAX6* gene, resulting in a nonsynonymous amino acid substitution (p.Arg26Trp). Both affected individuals exhibited severe CC accompanied by mild but consistent SPG symptoms. The p.Arg26Trp variant has previously been reported in two patients-one with CC and microcornea and another with CC and nystagmus-yet neither case included neurological manifestations such as SPG [10,11]. Furthermore, two additional substitutions at the same residue (p.Arg26Gly and p.Arg26Gln) have been associated with variable ocular phenotypes, including CC [12]. Notably, one individual carrying the p.Arg26Gly variant showed delayed speech and language development, suggesting possible neurological involvement [12].

*PAX6* encodes a transcription factor essential for eye development and is well known for its role in aniridia and related ocular defects [11,13]. It also regulates neural stem cell proliferation and differentiation [14-16]. Although *PAX6* variants have not previously been associated with inherited CC with SPG, a reported case of compound heterozygous truncating variants (p.Arg103Ter and p. Ser353Ter) exhibited severe neurological abnormalities, including hypertonia, suggesting a potential role in motor system dysfunction [17]. In our study, RNA sequencing of peripheral blood showed no significant difference in PAX6 expression between patients and controls. However, luciferase reporter assays demonstrated significantly increased reporter activity of the p.Arg26Trp variant compared with wild-type PAX6 (p = 7.93 × 10^-6^), suggesting altered transcriptional activity. Given the role of PAX6 as a transcription factor involved in both ocular development and neurogenesis [11,13-16], this altered activity may affect the transcriptional regulation of downstream targets and may contribute to the combination of ocular and neurological manifestations observed in the affected individuals. However, the precise molecular mechanism linking the p.Arg26Trp variant to SPG remains to be determined. While the possibility of additional undetected variants cannot be completely excluded, the functional alteration observed in our assay supports the potential pathogenic relevance of this variant.

## Conclusions

Collectively, our findings suggest that the p.Arg26Trp variant in PAX6 may represent a potential contributor to SPG in this family and may broaden the phenotypic spectrum of PAX6-related disorders. However, the mechanistic link between PAX6 and SPG remains uncertain, and the possibility of additional undetected variants cannot be completely excluded. Further studies, including replication in other families, are needed to clarify the contribution of PAX6 variants to SPG. These findings also highlight the importance of considering neurological involvement in patients with CC carrying *PAX6* variants.
